# To assess hemodynamic disturbances to the ostia of the renal arteries generated by the implantation of EVAR with a suprarenal fixation

**DOI:** 10.1097/MD.0000000000019917

**Published:** 2020-05-01

**Authors:** Lucie Salomon du Mont, Anne-Laure Parmentier, Marc Puyraveau, Frédéric Mauny, Benoit Guillon, Simon Rinckenbach, Patricia Costa

**Affiliations:** a Vascular and Endovascular Surgery Department, University Hospital Besancon; b EA3920, Université de Bourgogne Franche-Comté F-25000 Besançon; c Inserm CIC 1431, CHU Besançon, F-25000 Besançon; d Laboratoire Chrono-Environnement UMR 6249, CNRS, Université de Bourgogne Franche-Comté F-25000 Besançon; e Department of Cardiology; f Vascular Medicine Unit, Vascular and Endovascular Surgery department, University Hospital Besancon, France.

**Keywords:** aortic aneurysm abdominal, endovascular procedures, renal artery stenosis

## Abstract

**Introduction:**

The treatment of abdominal aortic aneurysm (AAA) is increasingly performed via endovascular aneurysm repair (EVAR). Different types of fixation are possible with EVAR, i.e., below (infrarenal fixation) or above (suprarenal fixation) the renal arteries. Hemodynamic alterations in renal arterial flow with suprarenal (SR) fixation remain to be demonstrated. The IFIXEAR (Impact of Supra-renal Fixation of EVAR on Hemodynamics of Renal Arteries) study is designed to assess the hemodynamic effects at the ostia of at least 1 renal artery, generated immediately post-surgery by the implantation of an aortic stent with SR fixation.

**Methods:**

IFIXEAR is a prospective, 2 center study. Every patient undergoing elective EVAR with SR fixation is eligible for inclusion. Patients with previous hemodynamic disturbances to the ostia of 1 of the renal arteries are not eligible. All patients undergo echocardiography and renal arteries duplex ultrasound within a month before surgery, and at 1 and 12 months after surgery. The primary endpoint is hemodynamic disturbance, defined as a peak systolic velocity greater than 120 cm/second, at the ostia of 1 of the renal arteries in the immediate postoperative period.

**Ethics and dissemination:**

The study was approved by the Ethics Committee “Comité de Protection des Personnes Ouest V” under the number 18/019-2 on April 20, 2018. All patients provide written informed consent before inclusion. The University Hospital of Besancon is the trial sponsor. Results of the study will be submitted for publication in a peer-reviewed international medical journal.

**Registration:**

The trial is registered with ClinicalTrials.gov (Identifier: NCT03594786, principal investigator: Dr Patricia Costa, Registered on April 24, 2018).

## 1 Introduction

Abdominal aortic aneurysm (AAA) affects 8% of men over 65 years of age. AAA rupture is fatal in more than 80% of cases. If detected early, prophylactic treatment, by either a surgical or endovascular approach, is recommended in France when the maximal transverse diameter exceeds 5 cm.^[1]^ Endovascular techniques are considered less invasive and are preferred in two thirds of cases.^[2,3]^ Endovascular aneurysm repair (EVAR) can be performed with infrarenal or suprarenal fixation (SR fixation). The choice depends the operators usual habits, the angulation ,and the length of the neck of the aneurysm.^[4]^

SR fixation presents at the level of its proximal part a bare metal stent, with in more than half of the cases, an interposition with regard to renal ostia.^[5,6]^ A hemodynamic impact is therefore possible at this level and likely to cause an alteration of renal vascularization. Animal studies have shown that SR fixation may compromise renal perfusion due to neointimal proliferation between the meshes of the uncovered stent, depending on the type of stent used.^[7,8]^ In addition, based on a computer simulation of patient-based data, Sun et al^[9]^ suggested a significant interference of SR fixation on renal arterial flow. They calculated that a single mesh of the covered stent centrally crossing the renal ostium could result in a flow reduction ranging from 21% to 29%, depending on the mesh size of the bare stent. However, in many studies, SR fixation has a worse effect in renal function. Elective EVAR is associated with a significant decline in estimated glomerular filtration rate (eGFR), and this effect is more pronounced with SR fixation.^[10,11]^ Nevertheless, the impact of SR fixation on renal artery function is not clearly defined.^[12]^ The development of renovascular disease, and possibly stenosis due to the stent crossing the renal ostia could explain this decline in renal function.

Currently, follow-up studies of EVAR are almost exclusively based on computed tomography angiography (CTA) scans.^[13]^ This exam does not appear suitable for assessing the renal hemodynamic impact of these patients.^[14]^ Duplex ultrasound is the only exam that can be used to perform hemodynamic analysis to detect flow disturbances in the renal arteries; and to appreciate the impact and the evolution of this disturbances and the stenosis.^[15]^ However, it is not used in this context for monitoring patients. In France, CTA scan is recommended at 1, 6, and 12 months after EVAR, and then every year thereafter, for follow-up.^[16]^ In the recent European recommendations, CTA scan is preferred at 1 month, the duplex ultrasound can be used in the follow-up then if the first CT scan is normal without endoleaks.^[4]^

Thus, the existence of hemodynamic alterations in renal arterial flow with SR fixation remains to be demonstrated.

## 2 Objectives

The IFIXEAR (Impact of Supra-renal Fixation of EVAR on Hemodynamics of Renal Arteries) study aims to describe the hemodynamic disturbances to the ostia of the renal arteries generated immediately postsurgery by the implantation of an aortic stent with suprarenal fixation.

The secondary objectives are to quantify the hemodynamic disturbances at the ostia of the renal arteries generated by the implantation of an aortic stent with suprarenal fixation at 1 year; to assess the proportion of patients with renal artery stenosis at 1 year; to assess the proportion of poststenotic turbulence at 1 year; and to evaluate the course of glomerular filtration rate before and at 1 year after surgery.

## 3 Methods and design

This manuscript is written in accordance with the SPIRIT guidelines.^[17]^

### 3.1 Study design

IFIXEAR is a prospective, two-center study. Patients are being included at the University hospital of Besancon and the University Hospital Dijon-Bourgogne.

### 3.2 Eligibility

Every patient undergoing EVAR with SR fixation is eligible for inclusion. Detailed inclusion and exclusion criteria for the present study are given below. Patients with ruptured AAA are not eligible for inclusion, because the management of the neck is different in emergency situations, and renal artery duplex ultrasound cannot be performed before the intervention.

### 3.3 Inclusion criteria

1. Signed written informed consent
2. Elective EVAR with suprarenal fixation
3. Women must be postmenopausal for at least 24 months, or surgically sterilized, or for women of childbearing potential, use of an effective method of contraception.
4. Affiliation to the French social security system, or beneficiary thereof.

### 3.4 Exclusion criteria

1. Peak systolic velocity (PSV) > 120 cm/second
2. Stenosis of at least 1 renal artery
3. Dialysis
4. Rupture of abdominal aortic aneurysm
5. Renal stenting during the procedure
6. Fenestrated EVAR
7. Open surgery
8. Legal incapacity or limited legal capacity
9. Subject in the exclusion period of another study
10. Pregnancy or lactation

### 3.5 Study outline

The eligibility criteria are verified during the preoperative consultation. The patient then receives information relating to the study by the surgeon and provides written informed consent for inclusion the day before the Duplex ultrasound.

Demographic data are collected. Patient characteristics, prior medical history, and preoperative creatinine levels are collected in the immediate preoperative period. Cardiovascular examination is performed. As part of this study, echocardiography to evaluate cardiac flow, and ultrasound of lower limbs and aorta are performed in the month before the intervention.

EVAR procedure is completed according to operator's usual habits without modification for the study.

At 1 month after EVAR, patients undergo CTA scan, provided renal function allows it, and a follow-up consultation with the surgeon. For the purposes of this study, patients will undergo (in addition to the recommended CTA scan) Doppler ultrasound of the renal arteries coupled with echocardiography to verify that renal artery hemodynamic changes are not secondary to modifications in cardiac output. Vascular exploration with ultrasound - Doppler will be performed by a single operator in each center, to guarantee better reproducibility and standardization of the examination. All ultrasound images taken for an examination will be recorded. Only hemodynamic impacts without a change in cardiac output will be attributed to suprarenal fixation of the stents.

At 1 year after EVAR, patients will undergo usual follow-up, and for the purposes of this study, will also undergo duplex ultrasound of the renal arteries and echocardiography. Creatinine levels will be recorded at 1 year.

### 3.6 Outcomes

The primary endpoint is significant hemodynamic disturbance in the immediate postoperative period, defined as peak systolic velocity (PSV) >120 cm/second at the ostia of at least 1 of the renal arteries, measured on duplex ultrasound at 1 month after intervention. The normal value of PSV is <120 cm/second in the renal artery.^[18]^ One examiner will perform the measurements in each center.

Secondary endpoints

1. Hemodynamic disturbances at 1 year, defined as a PSV > 120 cm/second at the ostia of at least 1 of the renal arteries.
2. Stenosis of 1 of the renal arteries at 1 year defined by at least 1 of these criteria:
  - PSV >180 cm/second
  - End-diastolic velocity (EDV) >50 cm/second
  - Renal-to-aortic ratio (RAR)>3.5 defined by relationship between PSV at the level of the lesion and the PSV of the interrenal aorta
  - Renal-to-renal ratio (RRR)>3.3 defined by relationship between PSV at the level of the lesion and the PSV of the renal artery downstream of the lesion
  - Stenosis revealed by hemodynamic disturbance: disappearance of the “notch” (pre-systolic) or increase of the systolic rise time or reduction of the resistance index or poststenotic turbulence
  - Morphologic signs of stenosis: decrease in the size of a kidney compared to the contralateral kidney or in reference to a previous exam.
3. One year hemodynamic repercussions on renal intra-parenchymal vascularization e.g., stenosis revealed by hemodynamic disturbance: disappearance of the “notch” (presystolic) or increase of the systolic rise time or reduction of the resistance index or poststenotic turbulence
4. Course of estimated glomerular filtration rate (in ml/minute/1.73 m^2^) over time, calculated using the CKD-EPI formula.^[19]^

### 3.7 Data management

Data will be managed using the Clean Web business application, an electronic clinical trial management solution from Telemedicine Technology. This solution meets the requirements of Good Clinical Practice, ICH, 21 CFR Part 11 (FDA) control and ensures secure data hosting. It also allows for a thorough implementation of users access rights based on their profile on the study.

### 3.8 Sample size calculation

The main objective was to evaluate the proportion of patients with hemodynamic disturbances. We assume that the maximum proportion of these patients is 20%. At an alpha risk of 5%, with an absolute precision of 10%, the number of subjects required is 62. We will include 70 patients to take into account any patients who are lost to follow-up.^[20]^

### 3.9 Statistical analysis

Quantitative variables will be described as mean ± standard deviation, or median [range] as appropriate. Qualitative variables will be described as number (percentage). The proportion of patients with hemodynamic disorders in the immediate postoperative period will be calculated with the associated 95% confidence interval. Preoperative and postoperative PSV variation as well as postoperative 1-year PSV variation associated with a 95% confidence interval will be calculated.

### 3.10 Monitoring

The Department of Research and Clinical Investigation of our institution will monitor all written informed consent, inclusion and exclusion criteria, and investigate all serious adverse events.

## 4 Ethics and dissemination

### 4.1 Ethical conduct of the study and informed consent

The French Ethics Committee (Comité de Protection des Personnes Ouest V, CHU de Rennes, Chairperson Pr Jean-Michel Reymann, N ° 18/019-2 on April 20 2018) approved this study. The IFIXEAR study is registered with ClinicalTrials.gov (Identifier: NCT03594786, principal investigator: Dr Patricia Costa, Registered on April 24, 2018). Eligible patients are screened during the presurgery consultation and receive all information relating to the study. Investigators obtain written informed consent from all patients before inclusion, the day before the Duplex ultrasound.

### 4.2 Planning and dissemination

Inclusions commenced on October 2, 2018. The planned duration of the trial is 2 years of inclusions, and a further year for follow-up (12-month follow-up of the last patient in). The university hospital of Besancon (CHU Besancon, Besancon, France) is the trial sponsor and the holder of all data. The results of the study will be submitted for publication in an international peer-reviewed journal and presented in abstract form in national and international conferences.

## Acknowledgments

The authors thank Fiona Ecarnot (EA3920, Department of Cardiology, University Hospital Besancon, France) for editorial assistance.

## Author contributions

**Conceptualization:** Patricia Costa, Lucie Salomon Du Mont and Simon Rinckenbach.

**Data curation:** Patricia Costa.

**Formal analysis:** Anne-Laure Parmentier and Marc Puyraveau.

**Investigation:** Patricia Costa, Lucie Salomon Du Mont, Benoit Guillon and Simon Rinckenbach.

**Methodology:** Anne-Laure Parmentier, Frédéric Mauny and Marc Puyraveau

**Supervision:** Lucie Salomon Du Mont, Anne-Laure Parmentier and Simon Rinckenbach.

**Validation:** Simon Rinckenbach.

**Visualization:** Patricia Costa, Lucie Salomon Du Mont, Anne-Laure Parmentier, Marc Puyraveau, Frédéric Mauny, Benoit Guillon and Simon Rinckenbach

**Writing – original draft:** Lucie Salomon du Mont and Patricia Costa.

**Writing – review & editing:** Patricia Costa, Lucie Salomon Du Mont, Anne-Laure Parmentier and Simon Rinckenbach.
